# Are the Derived Indexes of Peripheral Whole Blood Cell Counts (NLR, PLR, LMR/MLR) Clinically Significant Prognostic Biomarkers in Multiple Myeloma? A Systematic Review And Meta-Analysis

**DOI:** 10.3389/fonc.2021.766672

**Published:** 2021-11-23

**Authors:** Xinwen Zhang, Jialin Duan, Zhenyu Wen, Hao Xiong, Xiaomin Chen, Yang Liu, Kunyu Liao, Chunlan Huang

**Affiliations:** ^1^ Stem Cell Laboratory, Affiliated Hospital of Southwest Medical University, Luzhou, China; ^2^ Department of Hematology, Affiliated Hospital of Southwest Medical University, Luzhou, China; ^3^ Department of Orthopedics, Affiliated Hospital of Southwest Medical University, Luzhou, China

**Keywords:** multiple myeloma (MM), neutrophil-to-lymphocyte ratio (NLR), platelet-to-lymphocyte ratio (PLR), lymphocyte-to-monocyte ratio (LMR), monocyte-to-lymphocyte ratio (MLR), prognosis, meta-analysis

## Abstract

**Background:**

Multiple myeloma (MM) is an incurable malignant plasma cell tumor. Whole blood cell count (WBCC) derived indexes are widely used as a predictive biomarker for various types of solid and hematological malignant tumors. Our study is to evaluate its effectiveness in MM by meta-analysis.

**Methods:**

Relevant literatures were retrieved from PubMed, Embase and Web of Science databases according to PRISMA guideline. All relevant parameters were extracted and combined for statistical analysis.

**Results:**

Nineteen studies incorporating 3818 MM patients were eventually included in this meta-analysis. 13 studies evaluated that elevated NLR was significantly associated with poor survival outcomes (OS: HR=2.04, *P*<0.001; PFS: HR=1.96, *P*=0.003). Elevated NLR was revealed to correlate with ISS stage (ISS III VS I-II, OR=2.23, *P*=0.003). A total of 7 studies have shown that elevated LMR predicts a better prognosis in MM patients (OS: HR=0.57, *P*<0.001; PFS: HR=0.49, *P*<0.05), and two other studies demonstrated that increased MLR was related to poor OS/PFS (OS: HR=1.58, *P*<0.05; PFS: HR=1.60, *P*<0.05). However, in the other 6 studies including 1560 patients, the prognostic value of PLR had not been confirmed (OS: HR=0.89, *P*>0.05; PFS: HR=0.87, *P*>0.05).

**Conclusions:**

The indexes NLR and LMR/MLR derived from WBCC were validated to be useful biomarkers to predict the prognosis in MM patients, but the evidence of PLR was insufficient.

## Introduction

Multiple myeloma accounts for 1% of all cancers and about 10% of all hematological malignant tumors. It is characterized by a monoclonal proliferation of plasma cells and is associated with insufficient production of complete or incomplete immunoglobulins (1, 2). Unlike other malignant tumors that metastasize to bone, there is no new bone formation in osteolytic bone lesions of multiple myeloma (3). In the past 15 years, overall survival (OS) of multiple myeloma has improved significantly with the emergence of thalidomide, bortezomib and lenalidomide (4–7). More recently, drugs such as carfilzomib, pomalidomide and daratumumab have been approved for the treatment of relapsed multiple myeloma, further improving the efficacy (8, 9). With the continuous improvement of treatment level (including autologous hematopoietic stem cell transplantation and the emergence of new drugs), the life expectancy of MM patients is gradually prolonging (10, 11). However, almost all MM patients will relapse eventually, so we urgently need to find valuable prognostic indicators to assess the risk of patients and guide more active treatment, so as to delay the progress of the disease.

The International Staging System (ISS) divides the risk into three grades based on the concentrations of β2-microglobulin and serum albumin; which is related to the prognosis of patients with MM (12). In addition, the deletion of 17p13 (the locus for the tumor-suppressor gene, p53) leads to the loss of heterozygosity of *TP53*, which is considered to be a high-risk feature in MM (13, 14). Other high-risk chromosomal abnormalities in MM are characterized by structural changes, and several studies have confirmed that patients with t (4;14) and t (14;16) have a poor prognosis (15–17). However, MM patients with the same ISS stage also have different prognosis, and fluorescence *in situ* hybridization (FISH) is very expensive to evaluate the prognosis of MM patients. Therefore, we need to explore some convenient and easily available prognostic indicators. Inflammatory cells in the blood and systemic inflammatory response (SIR) have a considerable impact on the tumor microenvironment and the progression of malignant diseases, and are related to the prognosis of tumor patients (18, 19). A series of inflammation-related indexes derived from peripheral whole blood cell count (WBCC), including neutrophil-to-lymphocyte ratio (NLR), platelet-to-lymphocyte ratio (PLR), lymphocyte-to-monocyte ratio (LMR) or monocyte-to-lymphocyte ratio (MLR), are easily available and inexpensive, which are considered to be a kind of potential biomarkers.

At present, some studies of WBCC-derived indicators in MM have been reported, but the results of all publications are not consistent. Meta-analysis can overcome the difference of sample size between studies and calculate the best estimated value, which is a powerful statistical tool. The purpose of this study is to quantitatively describe the prognostic value of NLR, PLR and LMR/MLR in MM by meta-analysis.

## Material and Methods

### Search Strategy

We have conducted a comprehensive literature search of articles through the PubMed, Embase and Web of Science database, and there is no date limit for the search. The last search time is August 15, 2021. The main search terms included: “NLR” (e.g., “neutrophil-to-lymphocyte ratio”, “NLR”, “neutrophil lymphocyte ratio”), “PLR”(e.g., “platelet-to-lymphocyte ratio”, “PLR”, “platelet lymphocyte ratio”), “LMR”(e.g., “lymphocyte-to-monocyte ratio”, “LMR”, “lymphocyte to monocyte ratio”) or “MLR”(e.g., “monocyte-to-lymphocyte ratio”, “MLR”, “monocyte to lymphocyte ratio”) and “multiple myeloma” (e.g., “plasma Cell Myeloma”, “Kahler Disease”, “myelomatosis”). And we checked the relevant articles in the reference list.

### Inclusion and Exclusion Criteria

The inclusion criteria for this meta-analysis to select study are as follows: (i) studied patients with MM were diagnosed according to the criteria of the International Myeloma Working Group in 2014 (20). (ii) association between any of the WBCC-derived markers (including NLR, PLR, LMR or MLR) and overall survival (OS), progression-free survival (PFS) or other clinicopathological parameters was reported. (iii) the publication language of studies is limited to English. The exclusion criteria are as follows: (i) abstracts, letters, meta-analysis, case reports, or reviews. (ii) basic studies or animal trials. (iii) studies with insufficient data for estimating hazard ratio (HR) and 95% confidence interval (CI). (iv) patients suffered from other primary tumors or severe infections, or relapsed MM.

### Data Extraction and Quality Assessment

All candidate articles were evaluated and selected by two independent authors (Xin-wen Zhang and Jialin-Duan). Carry on the full-text analysis to the articles which cannot be classified only according to the abstract. If there is a disagreement, the two authors discuss and reach a consensus with the third author (Zhen-yu Wen). For each study, the following items were recorded: first author, year of publication, sample size, age, follow-ups, cut-off value, therapy, ISS stage and HRs with 95% CIs. In the absence of important data for the study, the correspondent author of the study was inquired by email. Two independent authors (Xin-wen Zhang and Jia-lin Duan) used the Newcastle-Ottawa Scale (NOS) to evaluate the quality of each included study (21). The NOS consists of three parts: the selection of subjects (0–4 points), the comparability between groups (0–2 points), and outcome assessment (0–3 points). NOS scores of ≥7 were assigned as high-quality studies.

### Statistical Analysis

We obtained HR and 95%CI from the final selected literature, as HR contains the influence of time-to-event outcomes, and can reflect the survival status of patients more reliably than other statistical measures (22). If HR is not explicitly provided in the study, the Engauge Digitizer (version 11.1) and method described by Tierney et al. was used to calculate HR from available statistics and Kaplan-Meier curves (23, 24). The combined odds ratio (OR) and its 95% CIs were used to evaluate the relationship between these derived indexes (NLR/PLR/LMR/MLR) and clinicopathological parameters. Cochran’s Q test and Higgins I-squared statistical methods were used to evaluate the heterogeneity of the included studies. A *P*_heterogeneity_< 0.10 or *I^2^* >50% suggested significant heterogeneity (25). Both the fixed-effects (Mantel–Haenszel method) model and the random effects (DerSimonian–Laird method) model were used to calculate the combined HRs and 95%CIs (26). Sensitivity analysis was used to evaluate the stability of merger results; subgroup analysis was used to explore the source of heterogeneity from the perspective of clinical heterogeneity and methodological heterogeneity. Publication bias was assessed by Begg’s funnel plot and Egger’s linear regression test (27, 28). All p-values were bilateral, p< 0.05 was considered statistically significant. All the data were analyzed by STATA12.0 software (STATA, Colleges Station, TX).

## Results

### Study Characteristics

**Figure 1:**
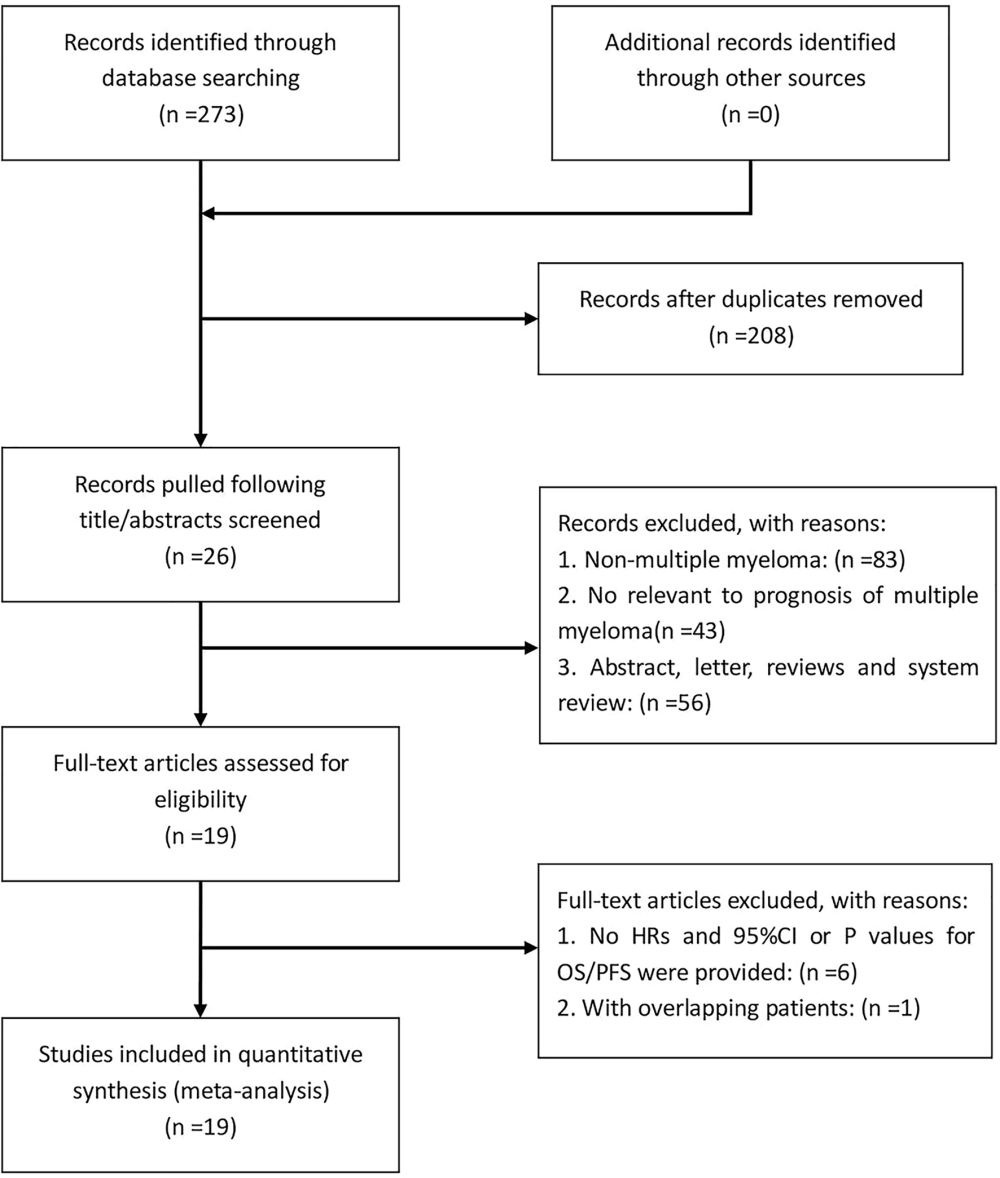
Flow diagram showed the selection process of studies for meta-analysis.

According to the established retrieval strategy, 273 potentially relevant studies were identified from the PubMed, Embase and Web of Science database. After removal of duplicates and browsing the titles and abstracts of the studies, 26 studies were assessed according to the inclusion and exclusion criteria. Seven other studies were subsequently excluded, and 19 retrospective studies with a total of 3818 patients published between 2013 and 2020 were finally enrolled in our meta-analysis (29–47). The process of literature screening is summarized in **Figure 1**. The NOS score of these 19 studies was ≥ 7, indicating that the risk of bias was low. Among them, 13 reported the correlation between NLR and the prognosis of MM patients, while only 7 and 9 studies were investigated in PLR and LMR/MLR, respectively. Participants in 10 studies were Asian, while in the other 9 studies were Caucasian. Fourteen studies reported HRs and 95%CIs directly, five of which calculated HRs by the Kaplan-Meier curves. 12 of these cohorts enrolled <200 patients and 7 cohorts had≥200 patients. A summary on the basic characteristics of included studies was listed in **Table 1**.

**Table 1:** Main characteristics of all the studies included in the meta-analysis.

Study	Year	Country	No. of patients (M/F)	Age (years)	Follow-up (months) (median and range)	ISS stage (n)	Cut-off value			Outcome	HR	NOS score
							NLR	LMR/MLR	PLR			
Witte HM (29)	2020	Germany	224(130/94)	59 (35–76)	72 (5–260)	I/II/III (121/63/40)	3	NR	150	OS/PFS	R(U)	8
Szudy-Szczyrek A (30)	2020	Poland	100	64 (53–69)	41.5	NR	2.86	NR	157.66	OS/PFS	R(M)	8
Yang Y (31)	2020	China	102 (67/35)	NR	14.23(0.17-60.4)	I/II/III (5/36/61)	NR	3.7	NR	OS	R(M)	8
Liu SW (32)	2019	China	175(95/80)	61	33.63(2.17–79.33)	I/II/III (23/44/108)	2	NR	NR	OS	R(M)	8
Sweiss K (33)	2019	USA	130(71/59)	59 (34–77)	25	I/II/III (16/25/55)	NR	5.7	NR	PFS	R(M)	9
Zou HQ (34)	2018	China	136(73/63)	61 (40–80)	27	I/II/III (14/106/16)	2	NR	NR	OS/PFS	R(U)	8
Zhou X (35)	2018	China	76(41/35)	63 (40–79)	34 (1–93)	I/II/III (3/35/38)	2.95	NR	NR	OS	R(U)	8
Solmaz S (36)	2018	Turkey	150 (58/92)	55 (26–70)	41	I/II/III (45/52/50)	1.46	0.27	120	OS	R(U)	9
Tian Y (37)	2018	China	285 (159/126)	NR	48 (2–84)	NR	NR	4.2	NR	OS/PFS	R(M)	7
Shi LH (38)	2017	China	560(344/216)	NR	64	I/II/III (100/195/265)	4	0.3	100	OS/PFS	R(U)	7
Romano A (39)	2017	Italy	208	58 (31–66)	36	I/II/III (54/77/77)	2	3.6	NR	PFS	R(U)	9
Onec B (40)	2017	Turkey	52(28/24)	65.5 (34–88)	35.1	I/II/III (7/18/27)	1.72	NR	NR	OS	R(M)	7
Li YJ (41)	2017	China	315(196/119)	NR	25 (1–64)	I/II/III (43/125/147)	2	NR	119	OS/PFS	R(U)	8
Kim DS (42)	2017	Korea	273(160/113)	64 (30–83)	NR	I/II/III (56/110/107)	2.25	NR	NR	OS	R(M)	7
Dosani T (43)	2017	USA	372(196/176)	67.3 (30–92)	37.5(1.16-152.9)	I/II/III (97/170/78)	NR	3.6	NR	OS/PFS	R(M)	7
Wongrakpanich S (44)	2016	USA	175	NR	2.78	I/II/III (46/61/34)	2.78	NR	155.58	OS	R(M)	7
Zhang XY (45)	2016	China	145(78/67)	NR	27 (2–96)	I-II/III (106/39)	NR	2.9	NR	OS	R(U)	7
Kelkitli E (46)	2014	Turkey	151(83/68)	63 (35–89)	41	I/II/III (23/54/74)	2	NR	NR	OS	R(U)	7
Shin SJ (47)	2013	Korea	189(98/91)	60 (29–84)	31.27(0.07-167.0)	I/II/III (35/87/61)	NR	2.9	NR	OS	R(M)	7

OS, overall survival; HR, hazard ratio, obtained by reporting in text (R). “M” means the HR come from multivariate analysis; “U” means the HR comes from univariate analysis; NR, not reported; NOS, Newcastle–Ottawa Quality Assessment Scale.

### The Prognostic Value of NLR

**Figure 2:**
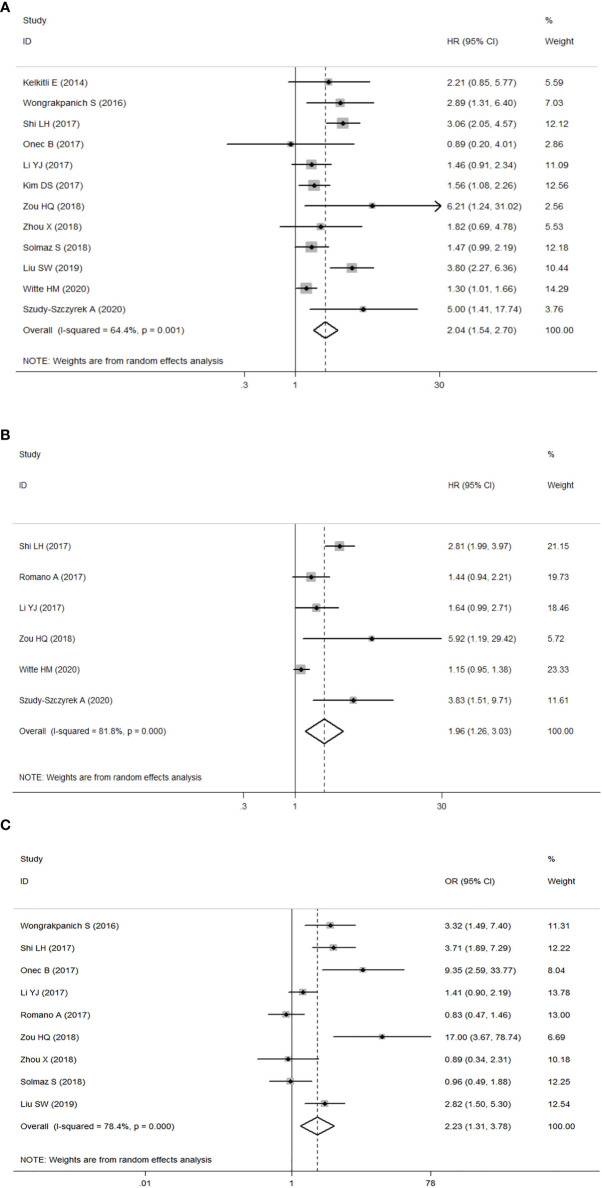
Forest plot for the association between neutrophil-lymphocyte ratio (NLR) and OS **(A)** /PFS **(B)** of patients with multiple myeloma (MM). **(C)** Forest plots of the association between NLR and ISS stage.

Thirteen studies evaluated NLR, of which 12 studies provided the data of elevated NLR and OS in MM patients. Though with significant heterogeneity (*I* ^2^ = 64.4%, *Ph*=0.001), we applied a random-effects model. The results showed that high level of NLR predicted a poor OS, with the combined HR of 2.04 (95%CI: 1.54-2.70, *P*<0.001; **Figure 2A**). Six studies reported the data of NLR and PFS in patients with MM. The combined HR obtained from random-effects model was 1.96 (95%CI: 1.26-3.03, *P*=0.003; **Figure 2B**), suggesting that MM patients with elevated NLR had a worse outcome for PFS. But the results also had significant heterogeneity (*I* ^2^ = 81.8%, *Ph*<0.001).

Then, we analyzed the relationship between NLR and clinicopathological parameters. Nine of the 12 studies provided data on NLR and ISS staging. The results showed that there was a significant correlation between high NLR level and advanced ISS staging of MM patients (ISS III VS ISS I-II: OR obtained from random-effects model: 2.23, 95%CI: 1.31-3.78, *P*=0.003; **Figure 2C** **)** with significant heterogeneity (*I* ^2^ = 78.4%, *Ph*<0.001).

In order to find out the source of heterogeneity, we conducted subgroup analysis of these 12 studies and 6 studies respectively, including study participants (Asian vs Caucasian), sample size (≥200 vs, <200), survival analysis (univariate analysis vs multivariate analysis), and cut-off value (2 vs not 2). The subgroup analysis did not alter the prognostic role of NLR in OS/PFS substantially, with significant heterogeneity across studies in all subgroups, as shown in **Table 2**. Then, meta-regression analysis was performed on 12 studies related to NLR and OS, but it still failed to explain the source of heterogeneity (with all *P*>0.05).

**Table 2:** Subgroup analysis for OS/PFS in MM patients with high NLR.

Analysis	N	References	Random-effects model		Fixed-effects model		Heterogeneity	
			HR (95%CI)	*P*	HR (95%CI)	*P*	*I* ^2^	*Ph*
OS	12	(29, 30, 32, 34–36, 38, 40–42, 44, 46)	2.040(1.541-2.701)	0	1.777(1.541-2.048)	0	64.40%	0.001
Subgroup 1: Univariate analysis	7	(29, 34–36, 38, 41, 46)	1.823(1.311-2.535)	0	1.633(1.381-1.930)	0	63.10%	0.012
Multivariate analysis	5	(30, 32, 40, 42, 44)	2.460(1.427-4.240)	0.001	2.218(1.693-2.907)	0	63.90%	0.026
Subgroup 2: Asian	6	(32, 34, 35, 38, 41, 42)	2.309(1.551-2.701)	0	2.196(1.785-2.703)	0	66.40%	0.011
Caucasian	6	(29, 30, 36, 40, 44, 46)	1.680(1.200-2.352)	0.003	1.473(1.212-1.790)	0	40.90%	0.133
Subgroup 3: Cut-off value=2	4	(32, 34, 41, 46)	2.576(1.372-4.839)	0.003	2.342(1.701-3.225)	0	65.50%	0.034
Cut-off value≠2	8	(29, 30, 35, 36, 38, 40, 42, 44)	1.857(1.360-2.534)	0	1.660(1.417-1.946)	0	62.50%	0.009
Subgroup 4: Sample size<200	8	(30, 32, 34–36, 40, 44, 46)	2.433(1.611-3.676)	0	2.236(1.732-2.887)	0	48.10%	0.061
Sample size≥200	4	(29, 38, 41, 42)	1.715(1.177-2.499)	0.005	1.603(1.351-1.902)	0	76.80%	0.005
PFS	6	(29, 30, 34, 38, 39, 41)	1.957(1.263-3.034)	0.003	1.478(1.280-1.707)	0	81.80%	0
Subgroup 1: Asian	3	(34, 38, 41)	2.410(1.460-3.979)	0.001	2.430(1.837-3.216)	0	52.70%	0.121
Caucasian	3	(29, 30, 39)	1.527(0.955-2.442)	0.077	1.236(1.045-1.462)	0.013	70.40%	0.034
Subgroup 2: Cut-off value=2	3	(34, 39, 41)	1.665(1.098-2.525)	0.016	1.606(1.166-2.211)	0.004	28.50%	0.247
Cut-off value≠2	3	(29, 30, 38)	2.515(0.996-4.645)	0.051	1.448(1.232-1.701)	0	91.80%	0
Subgroup 3: Sample size<200	2	(30, 34)	4.274(1.911-9.558)	0	4.274(1.911-9.558)	0	0.00%	0.645
Sample size≥200	4	(29, 38, 39, 41)	1.648(1.054-2.576)	0.028	1.427(1.233-1.652)	0	85.20%	0

N, number of studies; OS, Overall survival; HR, hazard ratio; 95% CI, 95% confidence interval; Ph, P values of Q test for heterogeneity test.

### The Prognostic Value of PLR

**Figure 3:**
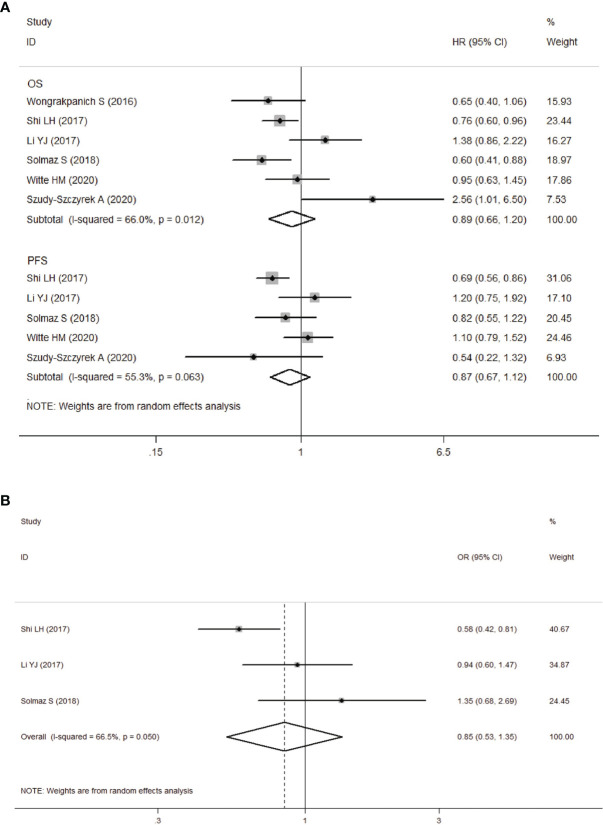
**(A)** Forest plot for the association between platelet-to-lymphocyte ratio (PLR) and OS/PFS of patients with multiple myeloma (MM). **(B)** Forest plots of the association between PLR and ISS stage.

Six studies including 1560 patients, revealed the relationship between PLR and OS, and five of them reported the data on PLR and PFS. We unified the experimental group of all the six studies into the higher PLR group, and the inconsistent studies need to re-calculate the reciprocal of HR and 95%CI. Then, the corrected results were included in the meta-analysis. Analysis of these six studies showed that there was no significant correlation between elevated PLR and OS in MM patients (HR obtained from random-effects model: 0.89, 95% CI: 0.66-1.20, *P*>0.05; **Figure 3A**), with significant heterogeneity (*I* ^2^ = 66.0%, *Ph*=0.012). In the other five studies with moderate heterogeneity (*I* ^2^ = 55.3%, *Ph*>0.05), combined by random-effects model had a HR of 0.87 (95%CI: 0.67-1.12, *P*>0.05; **Figure 3A**). Our results revealed that there was no significant correlation between elevated PLR and PFS in MM patients.

We next analyzed the relationship between PLR and clinicopathological parameters. Our meta-analysis showed that there was no significant correlation between PLR levels and ISS stages in MM patients (ISS III VS ISS I-II: OR obtained from random-effects model: 0.85, 95%CI: 0.53-1.35, P>0.05; **Figure 3B**), and the study was significantly heterogeneous (*I* ^2^ = 66.5%, *Ph*=0.05).

### The Prognostic Value of LMR/MLR

Seven studies involving 1431 patients reported the prognostic value of LMR in MM patients. The HRs and 95%CIs of all studies were corrected as the results of the higher LMR group (experimental group) before being included in the meta-analysis. Analysis of these 5 studies revealed that higher LMR was significantly associated with longer OS, and the heterogeneity between the studies was not significant (*I* ^2^ = 9.1%, *Ph*=0.355). A fixed-effects model was used for studies, with the pooled HR of 0.57 (95%CI: 0.45-0.72, *P*<0.001). Four studies reported the relationship between LMR and PFS in patients with MM. Our results showed that elevated LMR was significantly correlated with good PFS (HR=0.49, 95%CI: 0.42-0.57, *P*<0.05), with no heterogeneity (*I* ^2^ = 0%, *Ph*=0.892). Next, three studies showed that elevated LMR seemed to be more correlated with low-level ISS staging (ISS III VS ISS I-II: OR obtained from fixed-effects model: 0.68, 95%CI: 0.60-0.95, *P*<0.05), and there was no heterogeneity among the studies (*I* ^2^ = 11.1%, *Ph*=0.325).

**Figure 4:**
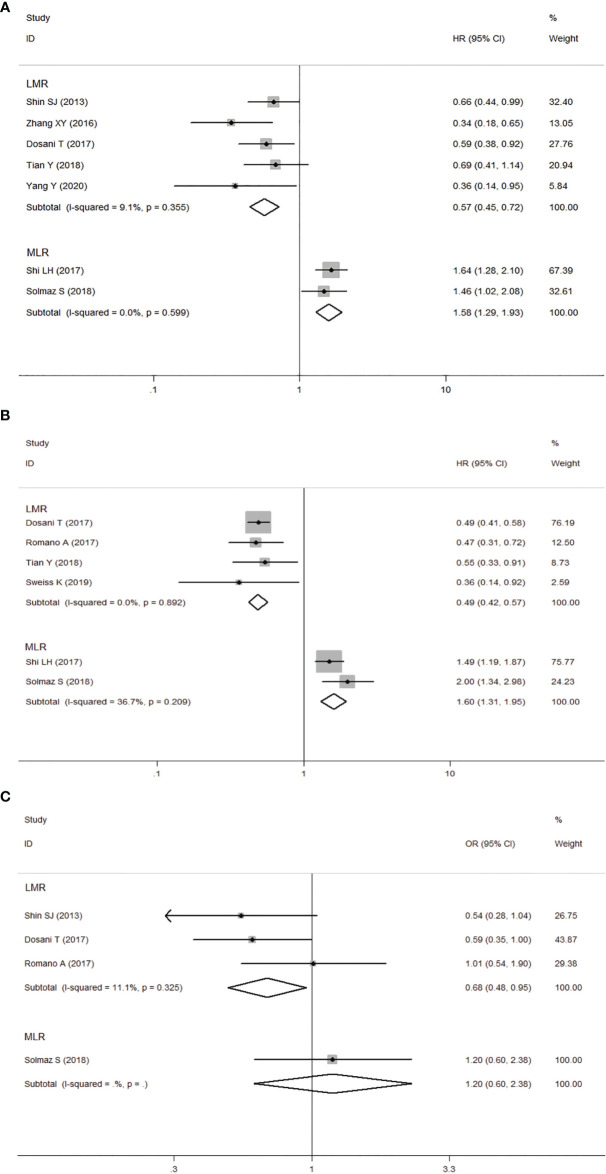
Forest plot for the association between lymphocyte-to-monocyte ratio (LMR) or monocyte-to-lymphocyte ratio (MLR) and OS **(A)** /PFS **(B)** of patients with multiple myeloma (MM). **(C)** Forest plot of the association between LMR/MLR and ISS stage.

Two studies provided data on both MLR and OS, PFS, the results revealed that higher MLR was significantly associated with shorter OS, PFS in MM patients (OS: HR=1.58, 95%CI: 1.29-1.93, *P*<0.05, *I* ^2^ = 0%, *Ph*=0.599; PFS: HR=1.60 95%CI: 1.31-1.95, *P*<0.05, *I* ^2^ = 36.7%, *Ph*=0.209). All results are shown in **Figure 4**.

### Sensitivity Analysis and Publication Bias

**Figure 5:**
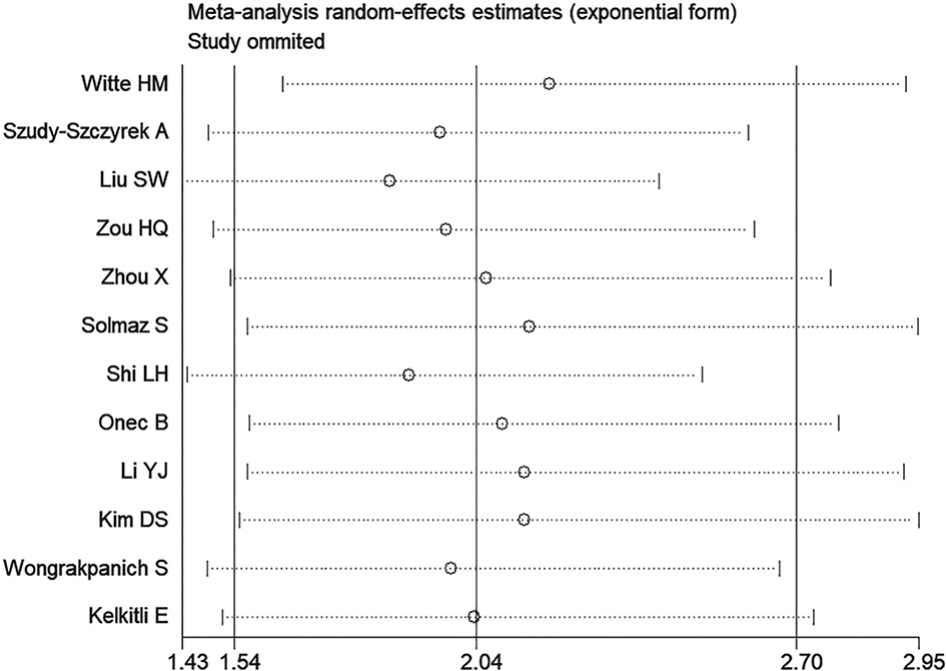
Sensitivity analysis of the association between neutrophil-lymphocyte ratio (NLR) and overall survival of multiple myeloma (MM).

We conducted a sensitivity analysis of NLR group and PLR group, deleting a single study each time to unveil the impact of the individual data set on the combined HRs. It was shown that the results of the combination of each group of studies were stable, and there existed no study that had a significant impact on the results (Only shown the results of NLR group for OS, **Figure 5**).

**Figure 6:**
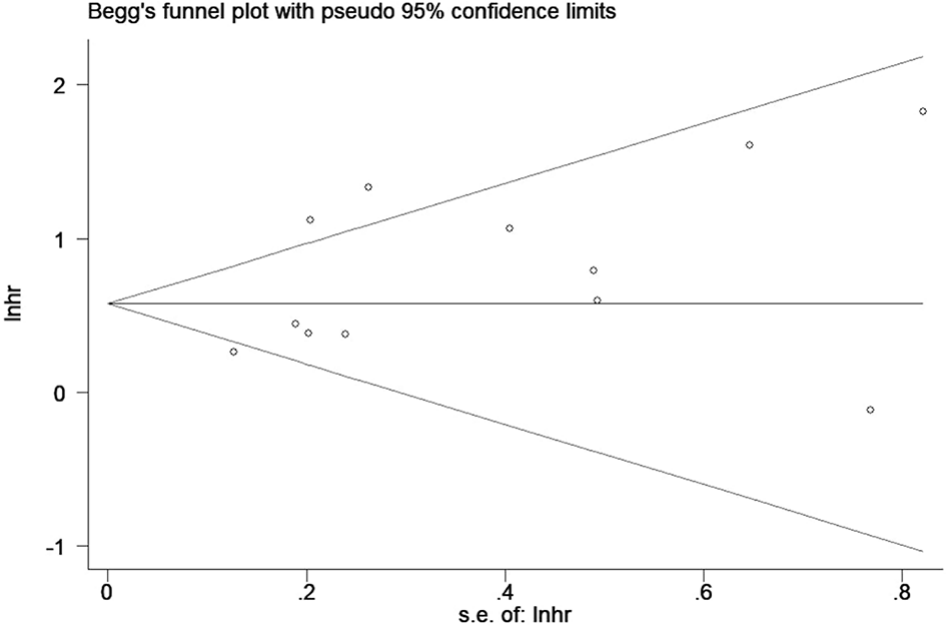
Begg’s funnel plots for detecting publication bias of the association between neutrophil-lymphocyte ratio (NLR) and overall survival of multiple myeloma (MM).

Publication bias was evaluated by Begg’s funnel plot and Egger’s linear regression test. The results showed that there was no significant publication bias among the included studies (Begg’s Test: *Pr*>|*z*|=0.304; Egger’s test: *P*>|*t*|=0.114). Begg’s funnel plots was shown in **Figure 6**.

## Discussion

Multiple myeloma (MM) is a malignancy of plasma cells, which is currently the second most common hematological malignant tumor (1). For newly diagnosed MM patients, the combination of proteasome inhibitors and/or immunomodulatory drugs combined with autologous stem cell transplantation (ASCT) can significantly improve the prognosis of MM patients (10, 11). However, multiple myeloma is still an incurable disease, and the clinical course is so highly variable. Therefore, we need to accurately and effectively evaluate the potential indicators which may be used to predict the prognosis of patients with multiple myeloma.

Multiple myeloma is strongly dependent on the bone marrow microenvironment, which can support the proliferation and survival of myeloma cells and is related to their drug resistance (48). With the deepening of the understanding of the tumor inflammatory microenvironment, we found that inflammation plays an important role in the occurrence, growth and development of tumors (18). Previous studies have shown that inflammatory markers are associated with the prognosis of different tumors, including non-small cell lung cancer, gallbladder cancer, diffuse large B-cell lymphoma, etc. (49–51). At present, we know that some inflammatory cells (including macrophages, dendritic cells, etc.) are involved in the coordination of MM microenvironment (52). Therefore, systemic inflammatory markers (NLR, PLR and LMR/MLR) derived from peripheral whole blood cell count (WBCC) have recently received close attention in MM.

Our meta-analysis showed that elevated NLR, decreased LMR/elevated MLR could predict poor OS/PFS in patients with multiple myeloma, but there was no significant correlation between PLR and prognosis of MM patients. The heterogeneity among the included studies may be partially contributed by study participants, cut-off value, survival analysis method and sample size. Since the number of studies included in the NLR group>10, we conducted a subgroup analysis. The results show that the effect of NLR on PFS may be related to cut-off value and sample size (there may be exist false positive results). However, the effect of NLR on OS was unaffected by the above factors. Although different treatments for MM patients may affect the outcome of OS/PFS in a single study, patients were grouped according to the pretreatment indicators. Therefore, the differences between treatment schemes should not be sufficient to have a significant impact on the results of meta-analysis.

Our findings inferred that NLR has significant prognostic value in MM patients, which is consistent with the studies of Mu et al. and Zeng et al. (53, 54). Our meta-analysis also showed that elevated NLR was closely related to advanced ISS staging. We know that ISS stage is based on serum albumin and β2-microglobulin, which is mainly used by the World Health Organization (WHO) to determine the prognosis of MM patients. High NLR reflects a decrease in the number of lymphocytes and an increase of neutrophils in tumor microenvironment. Absolute neutrophil count might serve as an important marker of systemic inflammation, which provides favorable environment for the occurrence and development of malignant tumors. On the contrary, the absolute count of lymphocytes reflects immunosuppression, which is associated with poor prognosis in a number of solid and hematological malignancies (55). In MM patients, cytokines including IL-6, IL-17, IL-21, IL-22 and IL-23 were detected at a high level (56). These inflammatory factors can combine with accessory cells in milieu, protect tumor cells from immune escape and simultaneously promote the development of tumors (57). These alterations of inflammatory components in the tumor microenvironment could be reflected by WBCC in a certain extent. Therefore, the elevated NLR generates a favorable immune microenvironment, which promotes vascular invasion and host immunosuppression, thereby correlating to poor prognosis of patients (58).

In our meta-analysis, 9 studies involving 2141 patients revealed the prognostic value of LMR/MLR in MM. The results showed that low LMR/high MLR predicted poor prognosis in MM patients. Studies have shown that circulating monocytes can produce a kind of tumor-associated macrophages (TAMs), which constitute an important proportion of tumor-related inflammatory cells (59). It is reported that TAMs are associated with poor prognosis of classical Hodgkin lymphoma (HL), follicular lymphoma (FL) and MM (60, 61). TAMs can be recruited to the tumor site by tumor-derived chemokines, thus affecting the number of peripheral blood monocytes. Therefore, the absolute monocyte count (AMC) in peripheral blood may reflect the recruitment degree of TAM to some extent (62). On the other hand, monocyte lineage cells are essential for innate immune response. A number of genes expressed by peripheral blood monocytes, whose products take part in immunologic response-2, and their expression levels are related to cancer prognosis. In addition, monocytes secrete TNF α and IL-1, thus monocytes play an important role in tumor microenvironment and could be used as markers of tumor load (63).

Our study showed that there was no significant correlation between pre-treatment PLR level and prognosis in MM patients. Solmaz et al. and Shi et al. both suggested that lower PLR is associated with poor survival in MM patients (36, 38). On the contrary, Szudy-Szczyrek et al. demonstrated that elevated PLR results in shorter OS (30). However, other studies suggested that PLR has not yet shown prognostic value in MM patients (41, 44). Current studies have confirmed that platelets can interact with platelet-derived growth factor and platelet factor to stimulate cell proliferation and metastasis through complex mechanisms of hemostasis activation as well as cell signal transduction, thus promoting tumor progression (64, 65). PLR combined with platelet and lymphocyte count index can more accurately reflect the pro-tumor efficacy and anti-tumor capacity of the host (66). The increase of PLR suggests that the balance of inflammation and anti-inflammation in the tumor may be disrupted, and the inflammatory response promotes tumor formation and is associated with a poor prognosis of some cancers (49, 67–69). This difference in multiple myeloma may be due to the pathology of the disease. Some studies have found that patients with MM show evidence of platelet activation, and the activated platelets secrete many kinds of cytokines that are required for the growth of myeloma cells, including IL-6, VEGF, SDF-1α and IGF-1, suggesting that platelets may affect the microenvironment (70). On the other hand, with the diffusion of malignant plasma cells in the bone marrow of MM patients, its aggregation can inhibit normal thrombopoiesis, and M protein produced in MM may adhere to the platelet surface, resulting in the decrease in the platelet activation and function (71). Based on our meta-analysis, we think that PLR could not predict the prognosis of MM patients at present. Though the treatment regimens of MM patients in the included studies are not consistent, which may lead to differences in the results between studies; in addition, the insufficient number of studies may lead to unstable results.

This meta-analysis still has some limitations that need to be carefully considered. Firstly, there is a significant heterogeneity between the studies included in NLR and PLR group. Despite the utility of sensitivity analysis and meta regression, the origin of heterogeneity could not be fully traced. Secondly, most of the studies are retrospective studies with low quality of evidence compared with other types of studies. Thirdly, the cut-off value of each study is not uniform, if each index can have a unified cut-off value standard, we may be able to better compare the differences between the studies. Finally, only few studies investigated PLR and LMR/MLR, so the statistical data of meta-analysis of the latter two indicators are under-powered. It is hoped that more with reasonably designed, high-quality, multicenter studies can be reported in the future to further enrich our results.

In summary, our meta-analysis validated that NLR and LMR/MLR derived from WBCC could be used as prognostic biomarkers to predict the survival outcome of MM patients, but the prognostic value of PLR is open to question. This series of indicators are inexpensive and easy to obtain, which help to assess the risk of MM patients and guide more active treatment, thus delaying the progress of the disease.

## Data Availability Statement

The data analyzed in this study is subject to the following licenses/restrictions: The original contributions presented in the study are included in the article/supplementary material. Further inquiries can be directed to the corresponding authors. Requests to access these datasets should be directed to CH, huangchunlan@swmu.edu.cn.

## Author Contributions

XZ, JD, and ZW designed the project. XZ, JD, and ZW performed data extraction and analysis. Quality assessment was conducted by HX, XC, YL, and KL. XZ and CH made critical changes to the manuscript and oversaw the project. All the authors read and approved the final manuscript.

## Conflict of Interest

The authors declare that the research was conducted in the absence of any commercial or financial relationships that could be construed as a potential conflict of interest.

## Publisher’s Note

All claims expressed in this article are solely those of the authors and do not necessarily represent those of their affiliated organizations, or those of the publisher, the editors and the reviewers. Any product that may be evaluated in this article, or claim that may be made by its manufacturer, is not guaranteed or endorsed by the publisher.
